# *Clostridium butyricum* Ameliorates *Salmonella* Enteritis Induced Inflammation by Enhancing and Improving Immunity of the Intestinal Epithelial Barrier at the Intestinal Mucosal Level

**DOI:** 10.3389/fmicb.2020.00299

**Published:** 2020-02-26

**Authors:** Xiaonan Zhao, Jie Yang, Zijing Ju, Jianmin Wu, Lili Wang, Hai Lin, Shuhong Sun

**Affiliations:** ^1^College of Animal Science and Technology, Shandong Agricultural University, Tai’an, China; ^2^Institute of Animal Science and Veterinary Medicine, Shandong Academy of Agricultural Sciences, Jinan, China

**Keywords:** *C. butyricum*, *Salmonella* enteritidis, immunity, intestine, intestinal microflora

## Abstract

This study was aimed to investigate the effects of *Clostridium butyricum* (*C. butyricum*) immunity and intestinal epithelial barrier function at the intestinal mucosal level, by using *Salmonella* enteritidis (*S.* enteritidis) to infect specific-pathogen-free (SPF) chickens and intestinal epithelial cells (IEC). We found that *C. butyricum* could decrease cytokine levels (IFN-γ, IL-1β, IL-8, and TNF-α) via the TLR4-, MyD88-, and NF-κB-dependent pathways in intestinal tissues and intestinal epithelial cells. Additionally, *C. butyricum* could attenuate bacteria-induced intestinal damage and increase the expression level of muc-2 and ZO-1 in the intestine and intestinal epithelial cells. Furthermore, *C. butyricum* altered the intestinal microbial composition, increased the diversity of the bacterial communities in the cecum of *Salmonella*-infected chickens. In conclusion, *C. butyricum* effectively attenuated inflammation and epithelial barrier damage, altered the intestinal microbial composition, increased the diversity of the bacterial communities in the intestine of *Salmonella*-infected chickens. The result suggests that *C. butyricum* might be an effective and safe therapy for the treatment of *Salmonella* infection.

## Introduction

*Salmonella* is a common bacterial entero-pathogen and one of the leading causes of serious illness in humans and animals, such as enteritis and diarrhea (Mathur et al., 2012). Over 20 million individuals suffer from typhoid fever, and more than 220,000 deaths each year have been reported around the world (Majowicz et al., 2010; Feasey et al., 2012).

Chickens have been recognized as an important reservoir for *Salmonella* (Chen and Jiang, 2014). The most frequently isolated serovar from chickens is *S.* enteritidis (Zhao et al., 2017). After oral ingestion in chickens, *Salmonella* initially breaches the epithelial lining, which is the first line of defense against the invasion of microbes and their associated lipopolysaccharide (LPS) and toxins. Impaired epithelial barrier function may predispose to various intestinal disorders, such as inflammation (Juan et al., 2018; Xiao et al., 2018). In addition, Mucins are the primary conpinents of intestinal mucus layer that are part of the innate immune system and act as a barrier against luminal pathologies (Forstner et al., 1995; Huang et al., 2015).

In recent years, antibiotics have been effectively used to treat *Salmonella* infection. Unfortunately, the widespread use of antibiotics has increased bacterial resistance and led to intestinal flora imbalance, which considerably diminish the efficacy of chemical antibiotics (Parry and Threlfall, 2008). Alternatively, the use of probiotic bacteria can modulate systemic and mucosal immune function, improve intestinal barrier function, alter gut micro-ecology, induce secretion of cytokines and Ig in serum, and perturb the MyD88 signaling pathway (Kusumawati et al., 2006; Shanahan, 2010; Madsen, 2012; Kemgang et al., 2014; Lim et al., 2017).

*Clostridium butyricum* is a gram-positive, obligate anaerobe and endospore-forming probiotic, which has been widely used for repairing intestinal epithelium, thereby improving gastrointestinal function (Cao et al., 2012). A preliminary study demonstrated that *C. butyricum* could reduce the colonization of pathogenic bacteria, weakening the inflammatory response (Zhang et al., 2016). However, the mechanism of protection remains to be elucidated.

In this study, we aimed to explore the mechanism by which *C. butyricum* could suppress the pathogenic strain *S*. enterica using the specific-pathogen-free (SPF) chicken model with an emphasis on the response at the intestinal mucosal level.

## Materials and Methods

### Ethics Statement

All procedures were approved by the Animal Care and Use Committee of Shandong Agricultural University (SDAUA-2016-016), and all husbandry practices and euthanasia were performed with full consideration of animal welfare.

### Bacterial Strains

*Clostridium butyricum* (AQQF01000149) was obtained from Dalian Sanyi Animal Medicine Company (China). The strain was cultured anaerobically with Reinforced Clostridial Medium (RCM) broth at 37°C for 48 h. According to the plate count method as described by Wei et al. (2013), the concentration of the bacteria was adjusted to 10^6^ colony forming units (CFU)/mL.

A virulent atrichia strain of *S.* enteritidis was obtained from the Avian Disease Centre of Shandong Agricultural University, and it was selected for the challenge study due to the invasive characteristic previously described (Zhao et al., 2017). The *S.* enteritidis strain was cultured with nutrient broth at 37°C for 12 h. To eliminate the possible LPS contamination, *S.* enteritidis cells were collected by centrifugation at 7,000 × *g* for 10 min and washed twice with PBS (pH 7.2), followed by dilution with PBS to a final cell count of 10^6^ colony forming units (CFU)/mL according to the LD50.

### Animals

Specific-pathogen-free chickens were obtained from Jinan SPAFAS Poultry Company (Jinan, China). SPF chickens refer to animals that do not have specific microorganisms or parasites, but may carry non-specific microorganisms and parasites, also known as third-class animals (The European Pharmacopoeia 7. 0,, 2010). Chickens were reared in the animal room of Shandong Agricultural University. Chickens were reared in metal cages, and the temperature was maintained at 30°C for the first 3 days and gradually reduced to 28°C during the last days of the experiment. Chickens were fed with a commercial diet and had free access to feed and water during the whole experimental period. The nutrient levels of the basal diet met the nutritional requirement of the chickens (NRC, 1994) (Table 1). At 1 and 7 days of age, birds were tested for the absence of *Salmonella* by taking cloacal swabs. Thereafter, a total of 60 health chickens were randomly assigned to three groups (*n* = 20/group): (Mathur et al., 2012) orally administered 0.2 mL sterile saline solution per chicken once every day from day 1 through day 14 [negative control group (NC)]; (Feasey et al., 2012) orally administered 0.2 mL sterile saline solution per chicken once every day from day 1 through day 14 and challenged with 0.2 mL *S.* enteritidis (10^6^ CFU/mL) on day 8 [*S.* enteritidis infected group, positive control (PC)]; and (Majowicz et al., 2010) orally administered 0.2 mL *C. butyricum* (10^6^ CFU/mL) once every day from day 1 through day 14 and challenged with 0.2 mL *S.* enteritidis (10^6^ CFU/mL) on day 8 [*C. butyricum* + *S.* enteritidis treatment (EXP)]. At the age of 14 days (6 dpi), all birds were euthanized via cervical dislocation. The tissues of duodenum, jejunum, ileum, and cecum were collected and stored in liquid nitrogen for mRNA and histological analysis. The cecal contents were collected and stored at −80°C for microbial composition analysis.

**TABLE 1:** The composition and nutrients of basal diet.

Ingredient	Content (%)	Chemical composition	Content
Corn	55.23	CP,%	20.90
Soybean meal	30.67	ME, Mcal/kg	3.00
Wheat shorts	4.00	Calcium,%	1.00
Fish meal	3.00	Total P,%	0.65
Soybean oil	2.90	Available P,%	0.45
DL-Methionine	0.27	Methionine + cysteine,%	0.90
NaCl	0.27	Lysine,%	1.05
Limestone	1.33		
Calcium phosphate	1.33		
Vitamin-mineral premix	1.00		

*^*a*^Crude protein content is 62.5% and metabolizable energy is 2.79 Mcal/kg. ^*b*^Metabolizable energy is 8.8 Mcal/kg. ^*c*^Supplied per kilogram of diet: vitamin A (retinyl acetate), 1,500 IU; cholecalciferol, 200 IU; vitamin E (DL-α-tocopheryl acetate), 10 IU; riboflavin, 3.5 mg; pantothenic acid, 10 mg; niacin, 30 mg; cobalamin, 10 ug; choline chloride, 1,000 mg; biotin, 0.15 mg; folic acid, 0.5 mg; thiamine 1.5 mg; pyridoxine 3.0 mg; Fe, 80 mg; Zn, 40 mg; Mn, 60 mg; I, 0.18 mg; Cu, 8 mg; Se, 0.15 mg.*

### Histological Study of the Cecum

One inch of the cecum of chickens was removed, fixed in 4% paraformaldehyde and prepared for histological studies as described by Sainte-Marie (1962). Paraffin sections of 5 μm were deparaffinized in xylene and stained with hematoxylin and eosin (H&E) for microscopic examination, and the overall quality of villi was observed.

### Microbial Composition Analysis

100 mg cecum contents samples were collected and microbial genomic DNA was extracted from cecum contents using TIANamp Stool DNA Kit (Tiangen, Beijing, China) according to the manufacturer’s instructions. The V4 hypervariable region of the 16S rRNA gene was amplified by PCR using 515F and 806R primers. Eighteen samples (*n* = 6/group) were sequenced on an Illumina MiSeq platform provided by Personalbio (Shanghai, China). Paired-end reads from the original DNA fragments were merged using FLASH. Clustering was performed using the UPARSE pipeline, and sequences were assigned to operational taxonomic units at 97% similarity (Schloss et al., 2009). The diversity and composition of the bacterial community was determined by α diversities according to Personalbio’s recommendations. The Chao1 and ACE indexes simply refer to the number of species in the community, regardless of the abundance of each species in the community, the Shannon’s diversity index considers both richness and evenness, the higher Chao1, ACE and Shannon index are, the higher the species diversity are.

### Real-Time PCR

Total RNA was extracted from duodenal, jejunal, ileal, and cecal tissues using Trizol reagent (Invitrogen, United States) according to the manufacturer’s instructions. Briefly, 50–100 mg tissue samples were ground to powder with liquid nitrogen and transferred to a tube with 1 ml of Trizol; after centrifuged at 4°C, 0.2 ml chloroform was added to the supernatant; after centrifuged at 4°C, the supernatant containing the intact RNA was transferred to a new tube, RNA was then precipitated with equal volume of isopropyl alcohol, and washed with 80% ethanol. The RNA was solubilized in RNase free water. RNA quantity and quality were evaluated using a NanoDrop^TM^ 2000 spectrophotometer (Thermo Fisher Scientific, Waltham, MA, United States), followed by cDNA synthesis via the Transcriptor First-Strand cDNA Synthesis Kit (Roche, China) using 2 μg RNA template. Real-time PCR was performed using SYBR Green I Master mix (Roche). Two microliters of cDNA, 5 μl SYBR Green buffer 2 × (Roche) and 2.5 pmol of each primer were combined for a total reaction volume of 10 μl. The thermocycler protocol consisted of a 5 min pre-incubation at 95°C for 20 s, 60°C for 30 s and 72°C for 20 s, and melt curves were added. The β-actin reference gene was chosen for the relative expression of targeted genes. mRNA relative expression was calculated using the 2^–△^^△^^Ct^ method. The primers used in this study are listed in Table 2.

**TABLE 2:** Primer sequences of targeted and reference genes.

Gene	Sequence (5′–3′)	References
TLR4	Forward: AGTCTGAAATTGCTGAGCTCAAAT Reverse: GCGACGTTAAGCCATGGAAG	Zhao et al., 2017
MyD88	Forward: TGATGCCTTCATCTGCTACTG Reverse: TCCCTCCGACACCTTCTTTCTA	Zhao et al., 2017
NF-κB	Forward: CAGCCCATCTATGACAACCG	Zhao et al., 2017
IFN-γ	Reverse: TCCCTGCGTCTCCTCTGTGA Forward: CTGACGGTGGACCTATTATTGTAG Reverse: GTTTGATGTGCGGCTTTGA	Zhao et al., 2017
IL-1β	Forward: GTGAGGCTCAACATTGCGCTGTA Reverse: TGTCCAGGCGGTAGAAGATGAAG	Zhao et al., 2017
IL-8	Forward: ATGAACGGCAAGCTTGGAGCTG Reverse: TCCAAGCACACCTCTCTTCCATCC	Zhao et al., 2017
TNF-α	Forward: TGCTGTTCTATGACCGCC Reverse: CTTTCAGAGCATCAACGCA	Zhao et al., 2017
Muc-2	Forward: AGGCCAGTTCTATGGAGCACAGTT Reverse: TTGAGTGCCCAGAGGGACATTTCA	Huang et al., 2015
ZO-1	Forward:GCGCCTCCCTATGAGGAGCA Reverse:CAAATCGGGGTTGTGCCGGA	Zuo et al., 2014
Occludin	Forward:TCGTGCTGTGCATCGCCATC Reverse:CGCTGGTTCACCCCTCCGTA	Zuo et al., 2014
β-Actin	Forward: GAGAAATTGTGCGTGACATCA Reverse: CCTGAACCTCTCATTGCCA	Zhao et al., 2017

### Primary Chicken Intestinal Epithelial Cell Culture

Specific-pathogen-free eggs were purchased from Jinan SPAFAS Poultry Company (China). Chicken intestinal epithelial cells (IECs) were prepared from 19-day-old SPF chicken embryos as described previously (Pierzchalska et al., 2012) with some modifications. Briefly, the duodenum was excised, cut into small pieces with a sterile scalpel blade, and dissected perpendicularly to expose the lumen. Small duodenal pieces were transferred to a tube filled with DMEM/Ham’s/F12 (Gibco, Grand Island, NY, United States) medium with 1% fetal bovine serum (Gibco), 50 μg/ml gentamycin (Invitrogen, Carlsbad, CA, United States), 100 μl/ml penicillin/streptomycin (10,000 U/ml/10,000 μg/ml) (Invitrogen, Carlsbad, CA, United States), 1 U/ml dispase II (Roche, Basel, Switzerland) and 75 U/ml collagenase (Gibco). Digestion was performed at 37°C under steady agitation for 2 h. The material was filtered, and larger pieces were discarded, while medium containing single cells and small pieces was centrifuged at 100 × *g* for 3 min. To separate mucus and IECs, a centrifugation step of 10 min was performed at 400 × *g*. Mucus covering the cell pellet was discarded. The remaining cell pellet was subsequently washed several times until the suspension was clear, and finally, 1 × 10^7^ cells were cultured in six-well plates and incubated at 37°C with 5% CO_2_. After incubation for 48 h, IECs were treated under three different conditions as follows: (NC) DMEM alone; (PC) *S.* enteritidis (10^6^ CFU) infection only; and (EXP) pre-incubation with *C. butyricum* (10^6^ CFU) for 2 h prior to exposure to *S.* enteritidis. At 2 and 6 h after *S.* enteritidis challenge, a portion of the cells were then collected and treated with lysis buffer to extract total RNA for real-time PCR.

### Statistical Analysis

Statistical evaluations were performed using a one-way ANOVA followed by a Duncan multiple range test or a Fisher least significant difference test using SPSS 16.0 (SPSS, Chicago, IL, United States). Data were visualized using GraphPad Prism 5 software (GraphPad Software, Inc., San Diego, CA, United States). *P* < 0.05 was considered significant.

## Results

### *C. butyricum* Improved Morphology and Integrity in the Cecum

**FIGURE 1:**
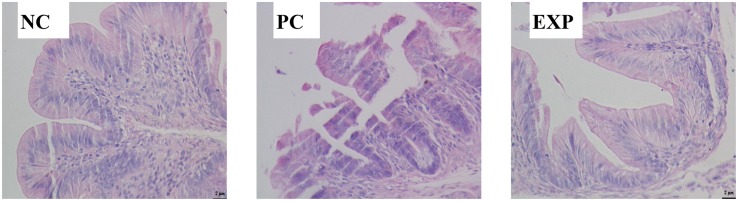
Representative histopathology of cecal tisssues at 6 days post-infection. Three independent experiments showing similar results with 6–8 chicken per treatment. NC, the negative control group; PC, the positive control group; EXP, *C. butyricum* + *S.* enteritidis treatment.

Microscopic examination revealed that chicken infected with *S.* enteritidis in the PC group showed surface damage and disruption to villi. Cecal tissue of chickens pre-treated with *C. butyricum* in the EXP group showed less severe surface damage to villi than did cecal tissue of chickens in the PC group. These observations demonstrate that pretreatment of *C. butyricum* resulted in a reduction of bacteria-induced intestinal damage (Figure 1).

### Determination of Cytokine Levels in Intestines

Cytokine levels were measured to test the hypothesis that early pretreatment of chicken with *C. butyricum* may alter cytokine production in intestinal tissue following *S.* enteritidis challenge.

**FIGURE 2:**
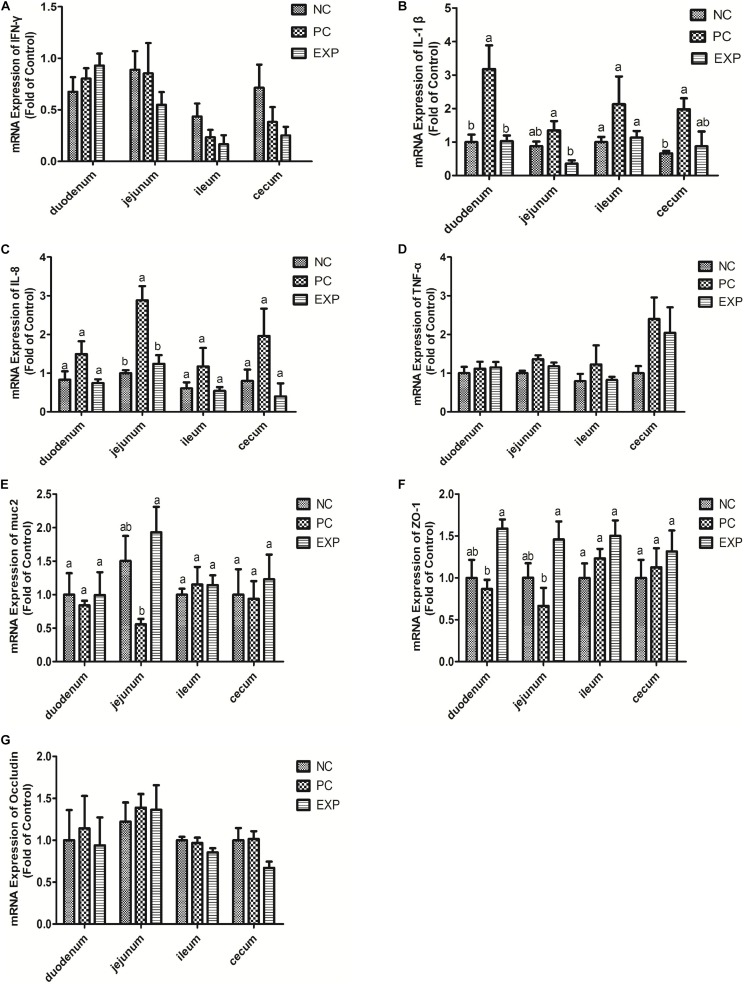
Expression level of cytokines (IFN-γ, IL-1β, IL-8, and TNF-α), muc2 mucin and the tight junction proteins (ZO-1 and Occludin) **(A–G)** in intestine tissues (duodenum, jejunum, ileum, and cecum) were estimated by real-time PCR. The bars represent the mean ± SD (*n* = 6/group). Different letters over the bars indicate statistically differences between the groups (*P* < 0.05), same letters over the bars indicate no statistically differences between the groups (*P* > 0.05). NC, the negative control group; PC, the positive control group; EXP, *C. butyricum* + *S.* enteritidis treatment.

**FIGURE 3:**
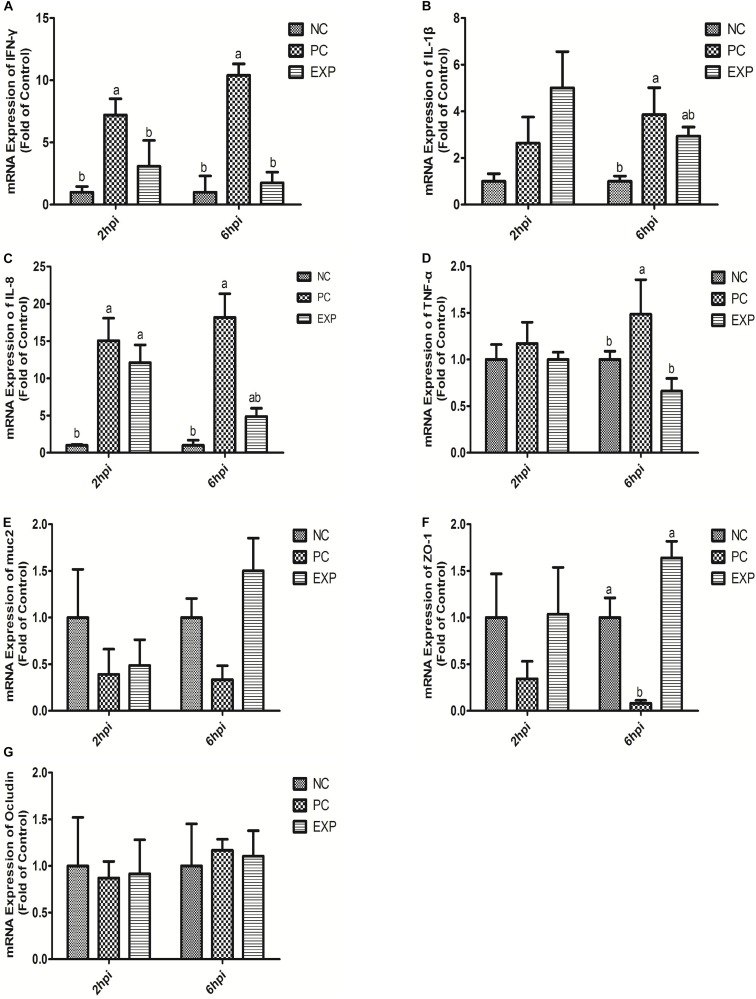
Expression level of cytokines (IFN-γ, IL-1β, IL-8, and TNF-α), muc2 mucin and the tight junction proteins (ZO-1 and Occludin) **(A–G)** in intestinal epithelial cells were estimated by real-time PCR. The bars represent the mean ± SD (*n* = 6/group). Different letters over the bars indicate statistically differences between the groups (*P* < 0.05), same letters over the bars indicate no statistically differences between the groups (*P* > 0.05). NC, the negative control group; PC, the positive control group; EXP, *C. butyricum* + *S.* enteritidis treatment.

The gene expression levels of cytokines IFN-γ, IL-1β, IL-8, and TNF-α in intestinal tissue (i.e. duodenum, jejunum, ileum, and cecum) were also evaluated. The results showed that at 6 days post-infection, no significant differences were found in IFN-γ and TNF-α among NC, PC, and EXP groups in intestinal tissue (i.e. duodenum, jejunum, ileum, and cecum) (*P* > 0.05) (Figures 2A,D). The gene expression level of IL-1β in the duodenum was significantly elevated in the PC group compared to the NC and EXP group (*P* < 0.05), but there was no significant difference between the NC and EXP groups (*P* > 0.05); in the jejunum, the gene expression level of IL-1β was significantly elevated in the PC group compared to the EXP group (*P* < 0.05), but there was no significant difference between the NC and EXP groups and the same change between NC and PC groups (*P* > 0.05); in the ileum, no significant difference of IL-1β was found among NC, PC, and EXP groups (*P* > 0.05); in the cecum, the gene expression level of IL-1β was significantly elevated in the PC group compared to the NC group (*P* < 0.05), but no significant difference was found between the NC and EXP groups and the same change between PC and EXP groups (*P* > 0.05) (Figure 2B). The gene expression level of IL-8 in the jejunum was significantly elevated in the PC group compared to the NC and EXP groups (*P* < 0.05), but no significant difference was found between the NC and EXP groups (*P* > 0.05); of note, no significant difference of IL-8 in duodenum, ileum, and cecum was found among NC, PC and EXP groups (*P* > 0.05) (Figure 2C). Furthermore, we investigated the effects of *C. butyricum* on cytokine expression in IECs *in vitro*. The results showed that after 2 h of infection, the expression level of IFN-γ was significantly elevated in the PC group compared to the NC and EXP groups (*P* < 0.05), but there was no significant difference between the NC and EXP groups (*P* > 0.05) (Figure 3A). The expression level of IL-8 was significantly elevated in the PC and EXP groups compared to the NC group (*P* < 0.05), but there was no significant difference between the PC and EXP groups (*P* > 0.05). Regarding the expression levels of IL-1β and TNF-α, no significant difference was found among the NC, PC, and EXP groups (*P* > 0.05) (Figures 3B–D). After 6 h of infection, the expression levels of IFN-γ and TNF-α were significantly elevated in the PC group compared to the NC and EXP groups (*P* < 0.05), but there was no significant difference between the NC and EXP groups (*P* > 0.05) (Figures 3A,D). The gene expression levels of IL-1β and IL-8 were significantly elevated in the PC group compared to the NC group (*P* < 0.05), but there was no significant difference between the NC and EXP groups, and the same change between PC and EXP groups (*P* > 0.05) (Figures 3B,C).

### *C. butyricum* Modulated muc2 Expression in Intestines of *S.* Enteritidis-Infected Chickens

The expression of muc2 in chicken intestines was detected via real-time PCR. The results showed that the expression level of muc2 in the jejunum was decreased in the PC group compared to the EXP groups (*P* < 0.05), but there was no significant difference between the NC and EXP groups, and the same change between PC and NC groups (*P* > 0.05). Of note, *C. butyricum* effectively attenuated the *S.* enteritidis-induced changes to muc2 expression in the jejunum. There were no significant differences in muc2 expression in the duodenum, ileum, or cecum among any of the groups (*P* > 0.05) (Figure 2E). Furthermore, we investigated the effects of *C. butyricum* on the muc2 expression in IECs *in vitro*, and our data showed that after 2 and 6 h post-infection, the gene expression level of muc2 was not significantly different among the different groups (*P* > 0.05) (Figure 3E).

### *C. butyricum* Increased Intestinal Barrier Function in *S.* Enteritidis-Infected Chickens

In this study, we evaluated the effects of *C. butyricum* on epithelial barrier function in the chicken intestines by detecting the expression level of Zonula occludens-1 (ZO-1) and Occludin via real-time PCR. The results showed that at 6 days post-infection, the expression level of ZO-1 in duodenum and jejunum was significantly decreased in the PC group compared with the EXP group (*P* < 0.05), but there was no significant difference between the NC and EXP groups, and the same change between PC and NC groups (*P* > 0.05). There were no significant differences in ZO-1 expression in either the ileum or cecum among any of the groups (*P* > 0.05) (Figure 2F). Similarly, no significant difference in Occludin levels was found in intestines among the NC, PC, and EXP groups (*P* > 0.05) (Figure 2G). We also investigated the effects of *C. butyricum* on tight junction (TJ) expression in IECs *in vitro*. The data show that after 2 h post-infection, the expression levels of ZO-1 and Occludin were not significantly different among NC, PC and EXP groups (*P* > 0.05) (Figures 3F,G); but after 6 h post-infection, the expression of ZO-1 was significantly decreased in the PC group compared to the EXP group (*P* < 0.05), and there was no significant difference between the NC and EXP groups (*P* > 0.05) (Figure 3F). The expression of Occludin 6 h post-infection was not significantly different among any of the groups (*P* > 0.05) (Figure 3G).

### *C. butyricum* Suppressed TLR4-, MyD88-, and NF-κB-Dependent Inflammation Pathways

**FIGURE 4:**
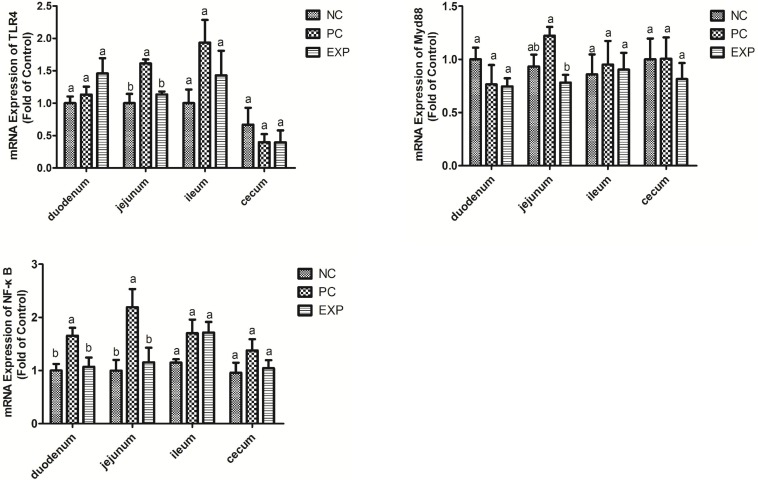
*Clostridium butyricum* supressed inflammation via TLR4-, MyD88-, and NF-κB-dependent pathways. Relative mRNA expression of TLR4, MyD88, and NF-κB in the intestine tissues (duodenum, jejunum, ileum, and cecum) were estimated by real-time PCR. The bars represent the mean ± SD (*n* = 6/group). Different letters over the bars indicate statistically differences between the groups (*P* < 0.05), same letters over the bars indicate no statistically differences between the groups (*P* > 0.05). NC, the negative control group; PC, the positive control group; EXP, *C. butyricum* + *S.* enteritidis treatment.

**FIGURE 5:**
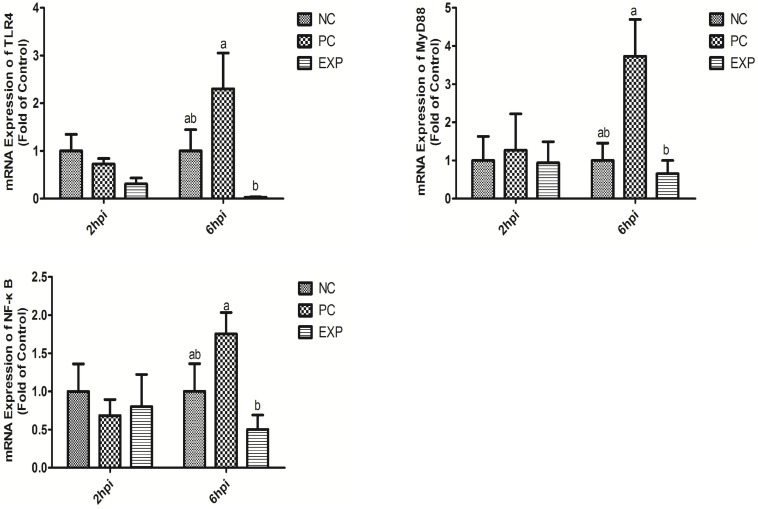
*Clostridium butyricum* supressed inflammation via TLR4-, MyD88-, and NF-κB-dependent pathways. Relative mRNA expression of TLR4, MyD88, and NF-κB in intestinal epithelial cells were estimated by real-time PCR. The bars represent the mean ± SD (*n* = 6/group). Different letters over the bars indicate statistically differences between the groups (*P* < 0.05), same letters over the bars indicate no statistically differences between the groups (*P* > 0.05). NC, the negative control group; PC, the positive control group; EXP, *C. butyricum* + *S.* enteritidis treatment.

Chickens in the EXP group had decreased gene expressions of TLR4, MyD88, and NF-κB in the jejunum compared to those in the PC group (*P* < 0.05), but there was no significant difference between the NC and EXP groups and the same change between PC and NC groups regarding the gene expressions of MyD88 (*P* > 0.05), which indicates a direct effect of *C. butyricum*. There were no significant differences in TLR4 and MyD88 expression in the duodenum, ileum, or cecum among any of the groups (*P* > 0.05). The expression level of NF-κB in duodenum was significantly elevated in the PC group compared with the EXP and NC groups (*P* < 0.05), but there was no significant difference between the NC and EXP groups (*P* > 0.05) (Figure 4). We further investigated the effects of *C. butyricum* on the TLR4, MyD88, and NF-κB expression levels in IECs *in vitro* and our results show that, after 2 h post-infection, the gene expression levels of TLR4, MyD88, and NF-κB were not significantly different among any of the groups (*P* > 0.05) (Figure 5); but after 6 h post-infection, *C. butyricum* decreased the gene expression levels of TLR4, MyD88, and NF-κB in the EXP group compared with the PC group (*P* < 0.05), but there was no significant difference between the NC and EXP groups and the same change between PC and NC groups (*P* > 0.05) (Figure 5).

### The Effects of *C. butyricum* on the Bacterial Community Within Chicken Cecum

**FIGURE 6:**
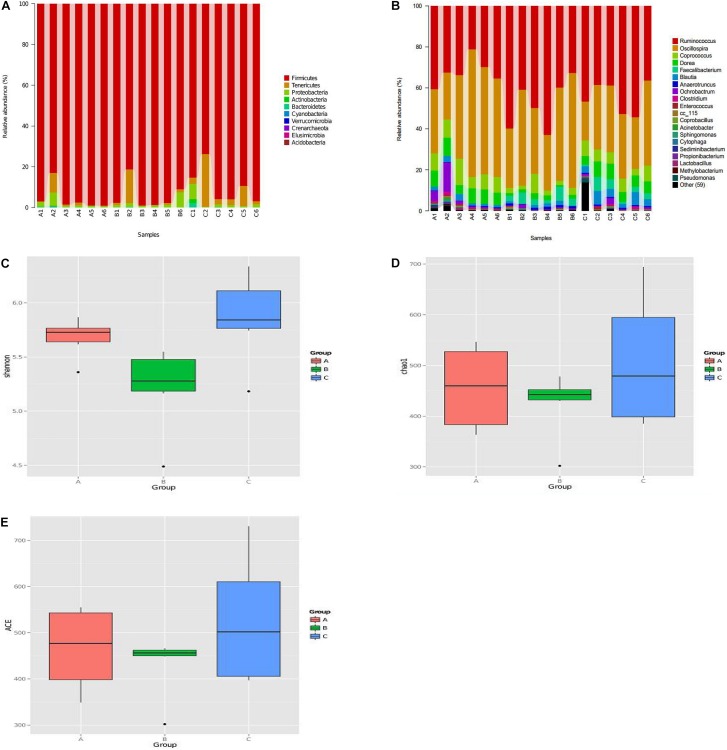
The diversity and composition of the bacterial communities were determined by α diversity according to Personalbio’s recommendations. **(A)** Relative abundance of the most abundant bacterial phyla. **(B)** Relative abundance of the most abundant bacterial genus. The diversity of the bacterial communities were determined by Shannon index **(C)**, Chao1 index **(D)**, ACE index **(E)**. **(A–C)** Represented sample of NC, PC, and EXP group. NC, the negative control group; PC, the positive control group; EXP, *C. butyricum* + *S.* enteritidis treatment.

We evaluated the effects of *C. butyricum* on the microbiota in chicken cecum using Illumina sequencing of the 16S rRNA V4 region. Firmicutes, Tenericutes, and proteobacteria were the three most abundant bacterial phyla in all samples, and *C. butyricum* increased the proportion of Tenericutes in the EXP chickens compared to the NC and PC groups (Figure 6A). The genera Ruminococcus, Oscillospira, Coprococcus, and Dorea were the most prevalent in all of the groups, and the proportion of Coprococcus and Dorea in NC and EXP groups was increased compared to the PC group (Figure 6B). The diversity of the intestinal bacterial community was determined by Shannon, Chao1, and AEC indices. The results show that *C. butyricum* increased the diversity of the bacterial community in the EXP group compared to the NC and PC groups (Figures 6C–E). Collectively, these data suggest that *C. butyricum* affects bacterial composition in the cecum of chickens.

## Discussion

Gram-negative *S. enterica* was identified as the most common cause of food poising in China (Ran et al., 2011) and is known to disrupt the intestinal epithelial layer during its infection (Coburn et al., 2007). In this study, *C. butyricum* protected the integrity of the villi in the cecum, limited the invasion of *Salmonella*; attenuated *Salmonella*-induced microbiota disruption in the intestine of chickens; improved intestinal epithelial barrier function through the modulation of Muc-2 and ZO-1 expression. Our results suggest that *C. butyricum* is a potential therapy for *Salmonella* infection or other intestinal diseases.

It has been reported that *Salmonella* could easily colonize the gut and induce a strong intestinal inflammatory response due to the defective microbial barriers and innate immune systems in the newly-hatched chicks (Brown et al., 2006). In the present study, *C. butyricum* significantly decreased the expression level of the pro-inflammatory cytokine (IL-1β and IL-8) production in intestines and the expression level of the pro-inflammatory cytokine (IFN-γ, IL-1β, IL-8, and TNF-α) in intestinal epithelial cells of chickens after *Salmonella* infection. The protective action of *C. butyricum* was similar to that of other probiotics (Castillo et al., 2013) and it maybe depended on its antibacterial acticity. Furthermore, we found that *C. butyricum* suppressed intestinal inflammation by downregulating the TLR4-, MyD88-, and NF-κB-dependent pathways in chickens with *Salmonella* infection, consistent with previous studies that *C. butyricum* can decrease pro-inflammatory cytokine levels by inhibiting the NF-κB signaling pathway in broiler chickens with *Salmonella* infection (Zhao et al., 2017). The result suggests the linkage of TLR4/NF-κB pathway may involved in the suppression of *C. butyricum* on *Salmonella* infection.

Muc2 is the major gel-forming mucin of the intestine and is the main structural component of the mucus gel. It is generally assumed that muc2 is essential for epithelial protection (Gill et al., 2011). In this study, muc2 production was decreased in the jejunum of chickens with *Salmonella* infection. However, *C. butyricum* attenuated the *Salmonella*-induced disruption of muc2 production, which is consistent with another study that showed supplementation of LGG before and after DON/ZEA exposure appeared to increase muc2 (Murphy et al., 2016), but our results are different than those reported in mice (Gaudier et al., 2005), that mucin gene expression was not altered by probiotic administration, this may be due to the differences in probiotic strains.

Tight junctions play a very important role in the intestinal mucosal barrier against macromolecular transmission (Ballard et al., 1995). ZO-1 and Occludin are important proteins responsible for the structural and functional organization of tight junctions (Fanning et al., 1998). In this study, we demonstrated that *C. butyricum* enhanced epithelial barrier function by increasing the expression of ZO-1 in intestinal tissue and IECs infected with *Salmonella*, which is consistent with a previous report showing that mRNA levels of ZO-1 in broiler chickens fed a 300 or 450 g/ton β-mannanase diet were significantly higher (Zuo et al., 2014).

Dietary supplementation of *C. butyricum* strains as a probiotic has become an effective alternative to the use of antibiotics to increase health and growth performance of chickens, as it has been shown that probiotics can positively affect the gut microbiota, which plays an important role in health and nutrient digestion in chickens (Yang et al., 2012). In this study, we found that *C. butyricum* treatment could alter the intestinal microbial composition and increase the diversity of the bacterial community, which could directly or indirectly impact chicken health and reduce or inhibit the presence of opportunistic pathogens and it may be due to its ability to produce metabolites, which can regulate the pH (acid change) of intestinal, inhibit pathogenic bacteria, and thus adjust the bacterial community structure. Our study aligns with another study that showed a diet supplemented with *Enterococcus faecalis* could shift microbial diversity in the porcine gut and inhibit pathogens (Li et al., 2016).

## Conclusion

*Clostridium butyricum* effectively attenuated inflammation and epithelial barrier damage, altered the intestinal microbial composition by increasing the diversity of the bacterial community, and promoted immune function in the intestines of *Salmonella*-infected chicken. *C. butyricum* might be an effective and safe therapy for *Salmonella* infection.

### Future Work

In future work, we will supplement the detection of *Salmonella* and *Clostridium butyricum* counts during the course of the experiments to further verify that the organism of the bacteria colonized the gut.

## Data Availability Statement

The datasets generated for this study are available on request to the corresponding author.

## Ethics Statement

The animal study was reviewed and approved by The Animal Care and Use Committee of Shandong Agricultural University.

## Author Contributions

HL, SS, and XZ conceived and designed the study. XZ, JY, ZJ, JW, and LW performed the experiments and analyzed the data. HL, SS, and XZ wrote and revised the manuscript.

## Conflict of Interest

The authors declare that the research was conducted in the absence of any commercial or financial relationships that could be construed as a potential conflict of interest.
